# Swarm-Intelligence-Centric Routing Algorithm for Wireless Sensor Networks

**DOI:** 10.3390/s20185164

**Published:** 2020-09-10

**Authors:** Changsun Shin, Meonghun Lee

**Affiliations:** 1 Department of Information and Communication Engineering, Sunchon National University, Jeollanam-do 57922, Korea; csshin@sunchon.ac.kr; 2 Department of Agricultural Engineering, National Institute of Agricultural Sciences, Jeollabuk-do 55365, Korea

**Keywords:** wireless sensor networks, swarm intelligence, AODV, routing algorithm

## Abstract

The swarm intelligence (SI)-based bio-inspired algorithm demonstrates features of heterogeneous individual agents, such as stability, scalability, and adaptability, in distributed and autonomous environments. The said algorithm will be applied to the communication network environment to overcome the limitations of wireless sensor networks (WSNs). Herein, the swarm-intelligence-centric routing algorithm (SICROA) is presented for use in WSNs that aim to leverage the advantages of the ant colony optimization (ACO) algorithm. The proposed routing protocol addresses the problems of the ad hoc on-demand distance vector (AODV) and improves routing performance via collision avoidance, link-quality prediction, and maintenance methods. The proposed method was found to improve network performance by replacing the periodic “Hello” message with an interrupt that facilitates the prediction and detection of link disconnections. Consequently, the overall network performance can be further improved by prescribing appropriate procedures for processing each control message. Therefore, it is inferred that the proposed SI-based approach provides an optimal solution to problems encountered in a complex environment, while operating in a distributed manner and adhering to simple rules of behavior.

## 1. Introduction

Swarm intelligence (SI) originates from the collective behavior of life groups [1]. It is a mechanism that can overcome the limitations of the perceptions of individual agents. SI deals with complex systems, in which individual agents interact with each other with minimal communication with neighboring agents. Owing to this property, SI has been applied to several engineering applications [2,3,4]. The SI-based bio-inspired algorithm shows features such as stability, scalability, and adaptability in an environment where many individuals exist, the environment changes dynamically, available resources are restricted, and objects with heterogeneous characteristics remain distributed and autonomous. This is similar to a communication network environment, along with its service requirements [5,6,7].

In this study, the ant colony optimization (ACO) algorithm—one of the most effective bio-inspired algorithms used in communication and networking technology—has been employed to address the limitations of wireless sensor networks (WSNs) [8,9,10,11]. Accordingly, an ACO-based WSN-routing algorithm has been proposed in this paper. The basic idea of the ACO algorithm is to provide the trailing ant with decision results from the leading ant, such that the trailing ant can use this information to identify an optimal solution [12,13,14]. The ant system is suitable for use in large dynamic systems, such as WSNs, for two reasons. First, within an ant colony, ants search for routes while exclusively using local information (i.e., the number of pheromones). Therefore, the ACO-algorithm-based system corresponds to a distributed control system used for communication over a wireless sensor network. Secondly, the ACO algorithm adapts well to unpredictable changes in the environment. In WSNs, an ACO-algorithm-based system is designed to reroute data according to the network traffic, thereby improving the performance of the entire transmission network. Therefore, using SI, an algorithm that maximizes the service life of a sensor network, while simultaneously distributing data traffic across the same, can be developed.

This paper presents the swarm-intelligence-centric routing algorithm (SICROA) for use in WSNs that aim to leverage the advantages of the ACO algorithm. The proposed algorithm considers each data packet transmitted to the base station as an ant, whereas each packet is considered the residual-energy pheromone of the sensor connected to the corresponding link upon selection of each successive hop in the path.

Additionally, SICROA ensures efficient energy utilization by each node, thereby maximizing the sensor network’s lifetime, while also allowing it to adapt to variations in the network environment [15].

Section 2 discusses the ACO and other applied algorithms that form the basis of the proposed algorithm. Section 3 presents the realization of collision avoidance via use of interrupts, link-quality prediction, and maintenance technologies incorporated within SICROA. Using simulation results, Section 4 compares the handling of the general routing problem by the proposed and other protocols, thereby demonstrating the superiority of the proposed algorithm. Finally, Chapter 5 discusses the simulation results, conclusions, and future work.

## 2. Related Works

### 2.1. Relevant Routing Algorithm for WSN

In WSNs, sink nodes are connected to multiple sensor nodes within a multi-hop network to acquire data from the sensor nodes. Because all sensor nodes are battery-powered and periodically generate a small amount of sensing data, their duration of continuous operation is limited. This problem can be addressed via the use of a data aggregation technique to enhance the energy efficiency of each sink node, thereby increasing the duration of continuous network operation [16,17,18]. In other words, the sensing information received from each node can be merged in the WSN relay node and delivered as a single dataset. For example, in the case of a WSN that measures the maximum/minimum temperature of a smart greenhouse, the relay node can be considered efficient when it exclusively transmits the said maximum/minimum temperature value from among several values received by multiple sensor nodes [19,20]. In several scenarios involving WSNs, the use of a data merging technique can reduce the data throughput and increase the network survival time. The authors in [21] present a routing method to be used in a WSN environment, where all of the sensor-collected data can be gathered at the sink node. This method was designed by considering in-network computational techniques, such as data merging, and, therefore, it was named “data-centric routing” [21]. This method corresponds to the existing address-centric routing scheme that aims to minimize the routing cost by reducing the distance between the source and destination nodes without considering in-network calculations, such as data aggregation. Therefore, data-centric routing aims to improve transmission or energy efficiency, instead of ensuring fast transmission, by considering data merging at intermediate nodes in the sensor network.

Data-centric routing in WSNs has been investigated to reduce the energy consumption of nodes or to increase the network survival time [22,23,24,25]. However, merging a large amount of data at intermediate nodes to reduce transmission-energy consumption causes a resultant increase in the waiting time. This increases the overall sensor-data-acquisition time at the sink node.

On the other hand, if an intermediate node transfers the collected data directly to the sink node without sufficient data merging, the resulting sensor-data-acquisition time can be reduced. However, this comes at the cost of an increase in the energy consumed by the node owing to an increase in the number of data transmissions. Therefore, a trade-off exists between the sensor-data-acquisition time and the energy consumed by the nodes.

### 2.2. ACO Algorithm

Ants are widely distributed around the world. While they are common insects, they have some unique capabilities. Specifically, one of the abilities of harvester ants is relying on a mechanism that controls the frequency of finding food. This is analogous to the function performed by a data management algorithm. Therefore, this phenomenon has attracted considerable research interest and triggered studies on optimization and control algorithms using ants’ swarm intelligence.

**Figure 1:**
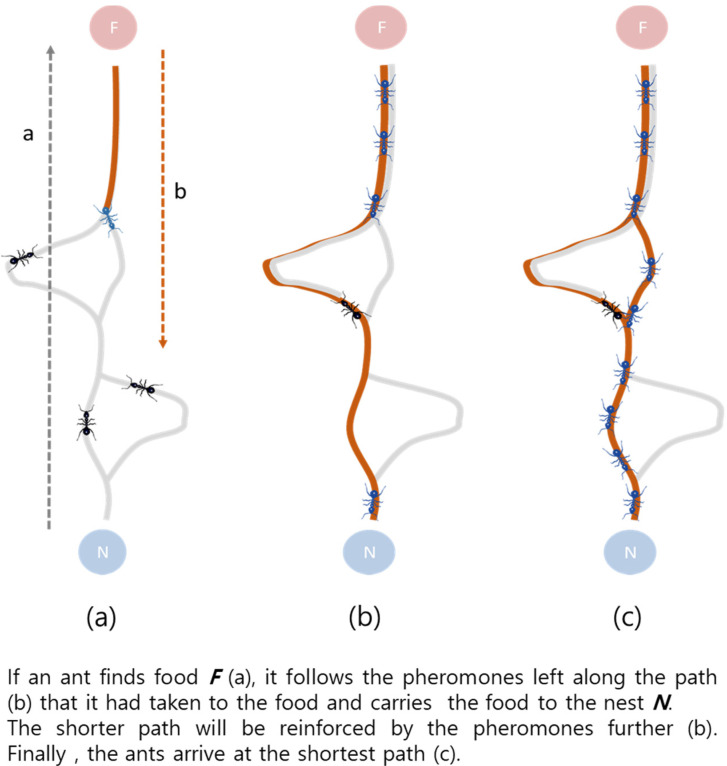
Ant colony optimization (ACO) algorithm.

The ACO algorithm, as proposed by M. Dorigo, is based on the cooperative behavior of ants attempting to identify the shortest path from their habitat to food [26]. It is used as a meta-heuristic approach to solve complex and difficult network problems, such as those pertaining to transportation and scheduling, store product displays, and optimized route search [27,28,29]. In the ACO algorithm, each ant acts as an artificial agent that attempts to solve its corresponding problem probabilistically. At each stage, the ants move and emit pheromones, as described in Figure 1. The said pheromones are updated during each iteration of the ACO algorithm, thereby increasing the probability of optimum-path calculation.

This study focuses on the indirect information transmission method using pheromones in the ACO algorithm [26]. Because a phase change due to the mobility of a sensor node causes a change in routing and multicasting paths, it is necessary to identify this change frequently for efficient data transmission [27,28,29]. However, a limited bandwidth or power supply issue makes the transmission of such information to each node difficult [30].

The ACO algorithm tries to mimic the behavior of an ant colony, wherein the blind worker ants transport food located far from their nest through the shortest path. The algorithm has been applied to various optimization problems related to communication networks, path exploration by a mobile object, scheduling, and job allocation [31,32].

As shown in Figure 1 [33], when an ant finds food (F), it transports it to its nest (N) leaving behind a pheromone trail (b) on its path (a). When a swarm of ants transport food through multiple paths, pheromones accumulate on the path with the shortest distance between the nest and the food. As ants tend to move along the path with the most pheromones, after a certain period of time, all of the ants end up transporting their food only through the shortest path. The amount of pheromone, $\tau_{ij}^{k}$, on the path, from *i* to *j*, after a certain period of time can be defined as follows:

(1) $$\tau_{ij}^{*}=(1-\rho )\tau_{ij}+\sum_{k=1}^{m}\Delta \tau_{ij}^{k}$$

In the above equation, $\tau_{ij}$ represents the current amount of pheromone on the path from *i* to *j*, ρ represents the evaporation rate of pheromones, m represents the number of ants moving from *i* to *j*, and $\Delta \tau_{ij}^{k}$ represents the amount of pheromone that the *k*-th ant releases along its path. An ant determines its next position based on the amount of pheromones (calculated as above); thus, it does not return to its own prior path, but selects a destination position within a one-hop distance. If the position that the *k*-th ant has not yet visited within the one-hop distance is defined as *l*, the set of these positions is defined as *N*( $S^{p}$), and the heuristic information is defined as $\mu_{ij}$, the probability $P_{ij}^{k}$ that the *k*-th ant moves to position *j* when it arrives at a position *i* is determined as follows:

(2) $$P_{ij}^{k}=\left\{ \begin{array}{c} \frac{\tau_{ij}^{\alpha }\mu_{ij}^{\beta }}{\sum_{c_{il}\in N(s^{p})}\tau_{il}^{\alpha }\mu_{il}^{\beta }}\\ 0 \end{array}\right.\begin{array}{l} c_{ij}\in N(s^{p})\\ otherwise \end{array}$$

In the equation, the heuristic information, $\mu_{ij}$, is defined as $\mu_{ij}$ = 1/$d_{ij}$ when the distance between *i* and *j* is $d_{ij}$; *α* and *β* are parameters that assign weights to the amount of pheromones and the amount of heuristic information, respectively. Therefore, if a path has more pheromones and is shorter, the probability of its use becomes higher.

## 3. Proposed SICROA

Because all nodes in a WSN environment are mobile, the network topology changes dynamically over time; the data transmission radius is limited by the available battery power; and it has an unstable routing path, where link disconnections occur frequently owing to interference, multipath fading, and/or collisions. To overcome these difficulties, this paper presents a biomimetic algorithm based on the SI-centric routing algorithm (SICROA) for WSNs. The said method is capable of providing an agile and appropriate response to routing problems.

### 3.1. Collision Avoidance through Interrupts (CATI)

The ad hoc on-demand distance vector (AODV)—a representative WSN protocol—defines a “Hello” message to alleviate frequent link-disconnection problems, and to allow mobile nodes on a path to exchange beacons with one another. However, link disconnections still frequently occur, and the transmission of periodic “Hello” messages can cause network congestion along with several other problems [34,35].

In ant colonies, individual ants passing through a path closer to the shortest one accumulate more pheromones. This accumulation of a large amount of pheromones along a path causes the ants to recognize it as the shortest path, thereby resulting in their movement along this recognized path.

Therefore, the aforementioned method results in collisions in the routing process, as well as a higher congestion density between adjacent nodes. Additionally, in the event of a traffic congestion, the establishment of a new path cannot be considered. This causes deterioration of the node-congestion state, increased end-to-end latency, and network-performance degradation.

To solve the aforementioned problems, collision avoidance through interrupts (CATI), wherein data relay is rejected by a node, which is considered to face increased traffic, by sending an interrupt message to the source node. Subsequently, the node that receives the message can establish a new bypass path. In addition, the node sending the interrupt message prevents a new path from being established through itself by not relaying the route request (RREQ) message to its neighboring nodes.

CATI replaces the “Hello” message with an interrupt to avoid collisions caused by periodic message transmissions, and it adopts the message formats of <Battery Check> and <Mobility Check> [36]. The <Battery Check> message checks the residual energy level. If the battery capacity is insufficient, or if the residual energy level of the node is less than or equal to the threshold value, <Battery Check> confirms the occurrence of the interrupt message by checking the threshold value. If the corresponding interrupt occurs, the status is transmitted to the neighboring node using the event process module. Because the neighboring nodes that receive this status can predict the disconnection of a link, the service life of the concerned node is set to zero in its corresponding route table, and the same is removed from the set of neighbor nodes after a certain time has elapsed.

**Figure 2:**
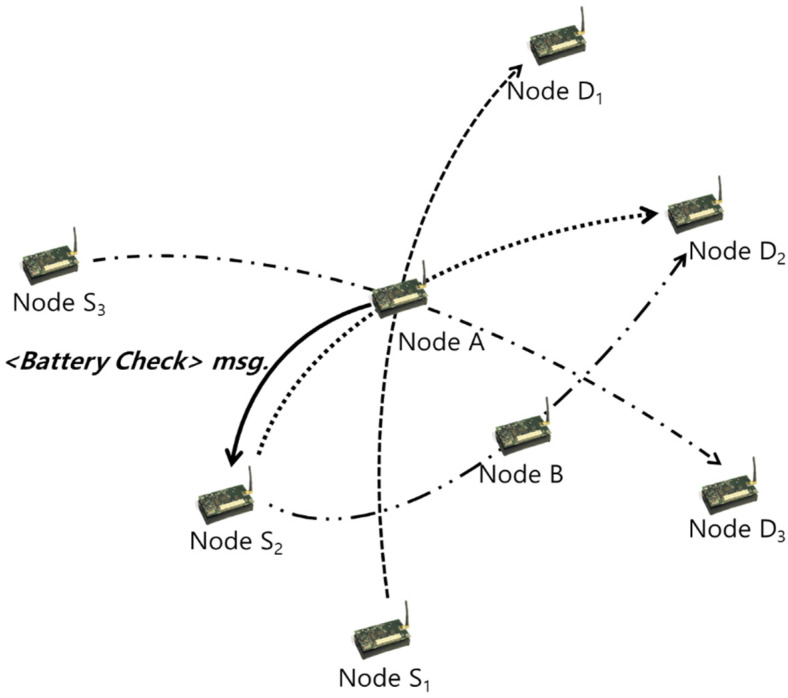
Collision avoidance through interrupts (CATI): <Battery Check> interrupt message.

Figure 2 depicts an example of the <Battery Check> interrupt. Node A denotes a relay node that exists on the data paths of the [S1, D1], [S2, D2], and [S3, D3] pairs. If node A predicts a link disconnection due to a low battery level caused by excessive traffic, the neighboring nodes receiving the message reduce the frequency of the link disconnections by modifying the received messages to bypass the path using alternate nodes prior to the occurrence of link disconnections.

Node S2, which receives the <Battery Check> message, initiates a new routing mechanism by broadcasting an RREQ message to establish a new route to node D2 [37]. If node S2 successfully completes the establishment of a new bypass route and initiates data transfer through the new route via node B, the route maintained by node A is no longer used. Therefore, the time for that entry expires, and it is naturally deleted from the routing table.

The <Mobility Check> message specifies the occurrence of a movement owing to changes in the position value of a node (A→A′). Consequently, the module that checks the movement status of the concerned node is called, and the corresponding module checks the coordinate values of the node at each period by setting a timer for transmitting the next <Mobility Check> message. Finally, when a change occurs, the event process module is used to send the status to neighboring nodes.

**Figure 3:**
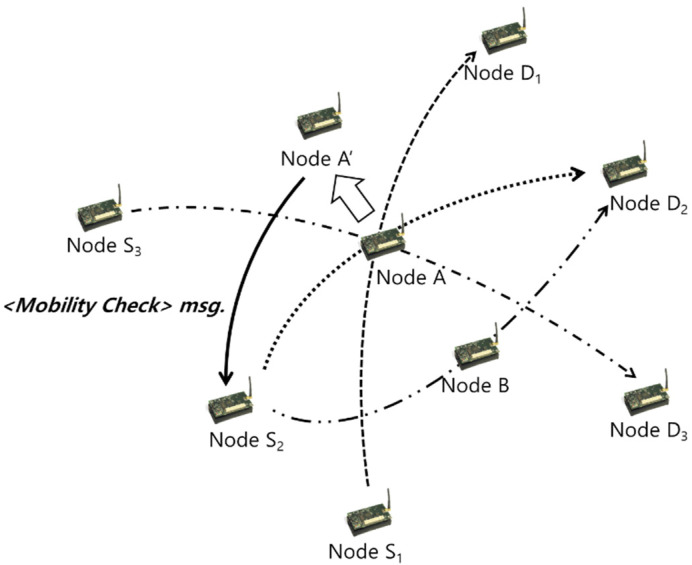
CATI: < Mobility Check> interrupt message.

The neighboring nodes receiving this status set the service life of the node in their route table at regular intervals, and whenever a <Mobility Check> message is received, the corresponding node in motion can be checked to verify if it lies within the transmission range. As depicted in Figure 3, when the link is broken owing to the sudden movement of the next hop or link disconnection during data transmission, path recovery is first performed using an alternate node to improve the frequency of path rescan.

Because CATI, which operates as presented in Algorithm 1, can determine the state of the neighboring nodes based on their type, the overall network performance can be improved by predicting or detecting link disconnections [33].
**Algorithm 1: Collision Avoidance through Interrupts*****[When node A receives a packet]*****if** RREQ message in a packet**if** node A is in the interrupt state for battery or mobilityignore the packet**else**process the packet using the existing routing algorithm**else if** receive interrupt message for battery or mobility**if** the interrupt state is destined to node Ainitiate the route-discovery mechanism of the existing routing algorithm**else**forward the packet with the alternative route
***[At the end of a time interval (for lifetime & mobility)]*****if** # of forwarding packets for the time interval ≥ thresholdchange the state to battery check or mobility checksend a <Battery Check> or <Mobility Check> message to the source of last received packet**if** # of forwarding packets for the time interval < thresholdchange the state to normal

### 3.2. Link-Quality Prediction and Maintenance

In SICROA, the route establishment and maintenance technology was applied by considering the residual energy of a node based on the AODV protocol. When setting the path by considering the residual energy of the node, it is possible to reduce the frequency of resetting the path that occurs owing to energy depletion. Furthermore, by setting the residual energy threshold of the node and notifying the source node before the path is disconnected, it is possible to reduce the data loss and transmission delay that occur owing to path resetting.

Reverse routing caused by the RREQ packet is similar to the flooding of existing AODV [38]. The source node broadcasts the RREQ packet to neighboring nodes. After the RREQ packet is received, the intermediate nodes store the route in their routing table and broadcast the RREQ packet to the neighboring nodes if they are not the destination nodes. If the intermediate node receives another RREQ packet through another path, the link dependency of the concerned path is verified. If the concerned path is link-independent, it is considered a potential alternative, and the received RREQ packet is discarded to ensure the link-independent path. Finally, when the destination node receives the RREQ packet, it also stores the path in its routing table prior to proceeding to the step wherein the forward path is established using the route reply (RREP) packet.

**Figure 4:**
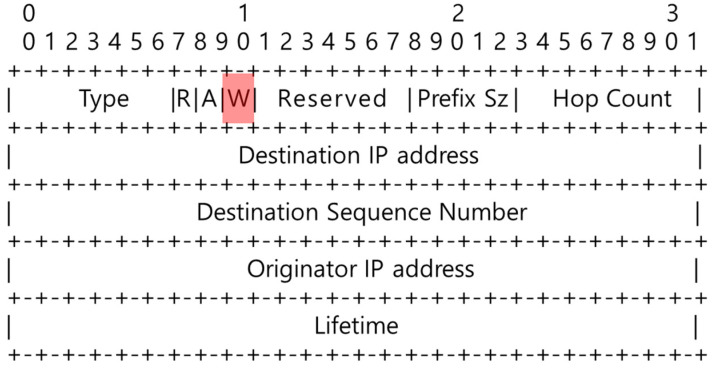
Route request (RREQ) message format of the swarm intelligence-centric routing algorithm (SICROA).

As illustrated in Figure 4, the *Warning_Energy* field, *W*, with a size of 1 byte was added to the RREP packet used in the existing AODV protocol. The destination node adds its minimum energy to the *Warning_Energy* field, and it subsequently sends the RREP packet to the unicast along the reverse path configured to receive the RREQ packet.

*Warning_Energy_t_ = Min (E_t_, Warning_Energy_t-1_)* (3)

The intermediate node that receives the RREP packet stores the route in its routing table. As described in Equation (3), *E_t_*, which denotes the current residual energy, is compared with the value stored in the *Warning_Energy_(t-1)_* field received from the previous node. Subsequently, the smaller value is stored in the current *Warning_Energy_(t)_* field, and the RREP packet is sent along the route stored in its routing table.

**Figure 5:**
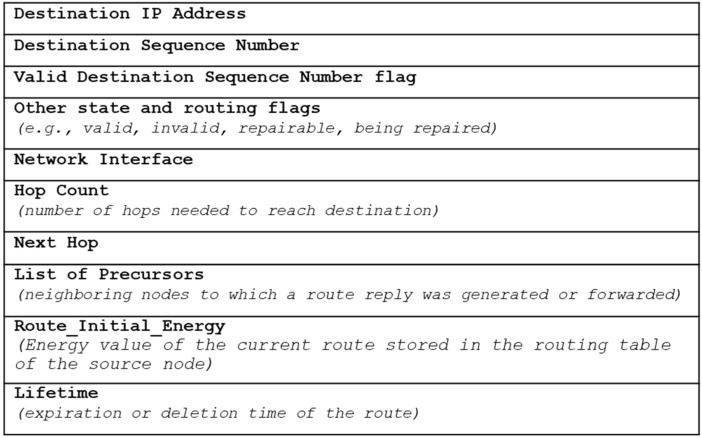
Route table entry when using SICROA.

When the source node receives the RREP packet, it saves the route and *Warning_Energy* value in its routing table, and initiates communication through the route. If the source node receives multiple RREP packets, it compares the value of the *Route_Initial_Energy* field of the current route stored in the routing node’s routing table with the value of the *Warning_Energy* field of the RREP packet. Subsequently, the communication continues by changing the path to that with a higher field value. If the *Warning_Energy* value remains unchanged or reduces, the existing path is used as it is. If the *Warning_Energy* value becomes larger, the path could be maintained for a longer duration, and thus, the resulting data transmission becomes stable. Figure 5 depicts the SICROA routing table, wherein the *Route_Initial_Energy* field is added to the existing AODV routing table.

## 4. Performance Evaluation

This section presents the results of the WSN simulations performed using Network Simulator-2 (NS-2), and compares them with those obtained using the proposed SICROA protocol, AODV, and dynamic source routing (DSR) routing protocols [39,40,41].

One hundred nodes were randomly distributed in a square region with 1000-m^2^ area, and the results obtained were measured in accordance with the change in the number of nodes in the proposed protocol. The experiment was repeated 10 times, and the number of nodes was increased from 10 to 100 in increments of 10 during each iteration. The random waypoint (RWP) was used as the mobility model, and the performance change was examined in terms of mobility by changing the maximum speed from 5 to 30 m/s.

During the 90-s simulation, the occurrence of data packets was observed every 0.25 s, and RREQ flooding occurred in intervals of 1, 2, and 3 s. ROUTE_TIMEOUT—i.e., the time for deleting the generated routing information—was set to 3 s, and the TTL value of all of the control packets was set to 20. To confirm the routing effect, they were only reflected in the results when the number of shortest-path hops between the origin and destination exceeded three. In addition, the TTL value was set large enough to ensure that RREQ and RREP remained flooded throughout the network during flooding for initial routing.

**Figure 6:**
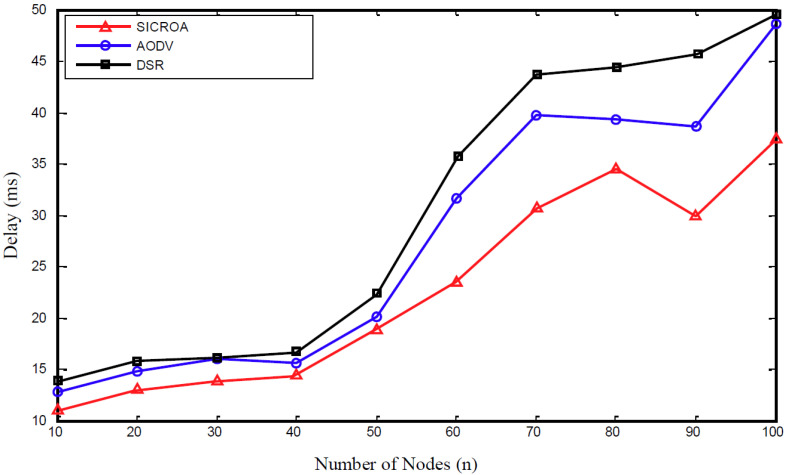
Latency measurement with respect to node count.

The end-to-end delay in Figure 6 denotes the time required for a data packet to arrive successfully at its destination, and it represents the length of the path used for data transmission. The proposed method demonstrates the lower delay performance compared with the existing AODV and DSR techniques. In addition, a lower delay performance is observed corresponding to a shorter flooding period at the beginning of the destination, owing to frequent updating of the new path. In general, for all of the routing protocols, the delay increases with the increase in mobility. However, in this study, the end-to-end delay of all of the routing protocols was reduced owing to the consideration of only successfully delivered data packets for delay-performance evaluation, and the new shortest path was found during path disconnection.

**Figure 7:**
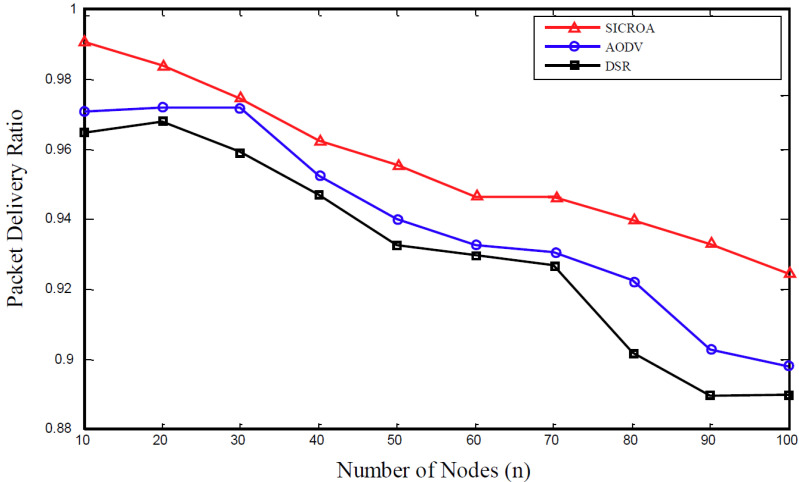
Packet delivery ratio with respect to number of nodes.

The packet delivery ratio in Figure 7 can be defined as the ratio of data packets arriving at the destination to the total number of data packets originating from the source. Thus, the packet delivery ratio is directly affected by the number of disconnections, because each node immediately discards the data packet, which needs to be delivered to the destination without resending it, when the path is disconnected. Therefore, the performance of the proposed method was observed to have greatly improved in terms of the packet delivery ratio.

**Figure 8:**
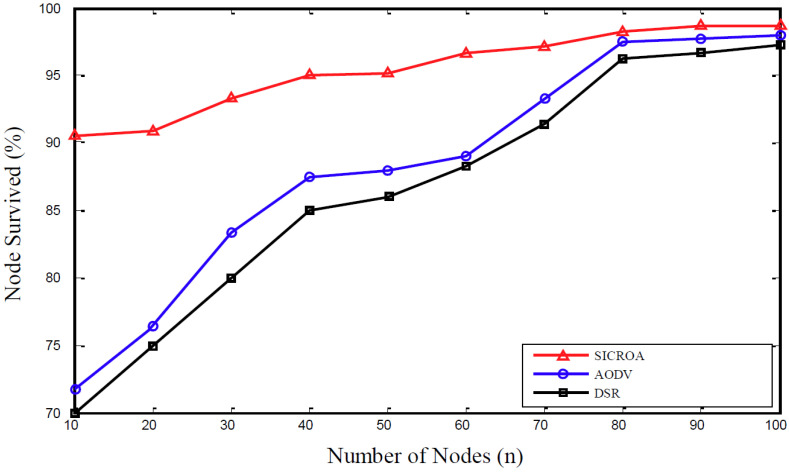
Changes in node survival rate with an increase in the number of nodes.

Figure 8 shows the trend concerning changes in the survival rate of nodes with an increase in the node count until the end of the simulation. The observed trends correspond to the formation of a large network. As can be confirmed, SICROA demonstrates a higher survival rate, owing to its consideration of residual energy, which is not considered by other routing protocols. Upon selection of the network path, both the transmission and reception of the control packets is reduced, which in turn reduces energy consumption.

Based on the observed trends concerning the simulation factors, as described in Figure 6, Figure 7 and Figure 8, the proposed SICROA routing protocol clearly outperforms AODV and DSR. Because the transmitting node exchanges paths through the researching path prior to the occurrence of node failure and link damage, no data or link loss occurs. Additionally, the packet delivery rate in SICROA exceeds those observed when using DSR and AODV. In other words, because the transmitting node continues to initiate path discovery prior to packet transmission, the disconnection of all paths can be avoided. Any increase in the average node speed, number of connections, or number of nodes tends to reduce the packet delivery rate, and this can be attributed to the high node mobility that causes frequent link damages, owing to which, several packets are discarded. Additionally, the increase in connection and node counts causes an increase in signal-strength traffic interference, which in turn increases the possibility of link damage.

## 5. Conclusions

The proposed routing protocol overcomes the disadvantages of AODV and improves routing performance via the incorporation of collision avoidance, link-quality prediction, and maintenance techniques that mimic the ACO algorithm. SI can operate reliably in accordance with simple rules of behavior, while providing reliable solutions to given problems in complex environments.

In this study, the performance of the routing protocol was improved by replacing the periodic “Hello” message with an interrupt message capable of detecting and predicting link disconnections. As observed, routing performance can be further improved via the addition of processing procedures for each message type. In addition, when the signal strength of a received data packet approaches the residual-energy threshold prior to energy loss, an increase in path-loss tendency can be observed. In contrast, when the signal strength of a received packet falls below the minimum threshold, a pre-warning packet is generated. Simulation results reveal that the proposed method is superior to the existing AODV and DSR routing protocols. The trade-off between the acquisition time of the sensing information and the energy consumed by the nodes is well resolved. In addition to providing a highly reliable and robust path for information transmission, the proposed method improves source-to-destination data latency, thereby reducing the frequency of the link disconnections and unnecessary control packet transmissions within the network.

In view of its above-mentioned features and advantages, the proposed biomimetic algorithm is expected to be effectively utilized in large-scale communication networks. This requires sophisticated mathematical modeling of various biological systems, as well as rigorous performance verification based on several system-environment variables. In future, further research is required on techniques to maintain a continuous alternative path in the proposed algorithm and to increase the reliability of the path in one-way links that often occur in wireless network environments. In addition, some path improvements can be made, such as using the results of the proposed method to compare with other methods (e.g., recursive neural networks).
